# Moyamoya associated with Turner syndrome in a patient with type 2 spinocerebellar ataxia—Occam’s razor or Hickam’s dictum: a case report

**DOI:** 10.1186/s12883-022-02912-x

**Published:** 2022-10-08

**Authors:** Paulo Ribeiro Nóbrega, Francisco Bruno Santana da Costa, Pedro Gustavo Barros Rodrigues, Thais de Maria Frota Vasconcelos, Danyela Martins Bezerra Soares, Jéssica Silveira Araújo, Daniel Aguiar Dias, Manoel Alves Sobreira-Neto, Anderson Rodrigues Brandão de Paiva, Pedro Braga-Neto, Fernando Kok, Eveline Gadelha Pereira Fontenele

**Affiliations:** 1 Division of Neurology, Department of Clinical Medicine, Federal University of Ceará, Rua Prof. Costa Mendes, 1608 - 4° andar - Rodolfo Teófilo, Fortaleza, Ceará Brazil; 2 Center of Health Sciences, Ceará State University, Fortaleza, Brazil; 3 Division of Endocrinology and Metabology, Department of Clinical Medicine, Federal University of Ceará, Fortaleza, Brazil; 4 Division of Radiology, Federal University of Ceará, Fortaleza, Brazil; 5 Department of Neurology, São Rafael Hospital, Rede D’Or São Luiz, Salvador, Brazil; 6 Department of Neurology, Clinics Hospital of the University of São Paulo Medical School, São Paulo, Brazil; 7 Mendelics Genomic Analysis, São Paulo, Brazil

**Keywords:** Spinocerebellar ataxia, Metabolic and endocrine disorders, Genetic and inherited disorders, Chromosome disorders, Cerebrovascular malformations, Cerebrovascular diseases and cerebral circulation, Angiography

## Abstract

**Background:**

Turner syndrome (TS) is a rare condition associated with a completely or partially missing X chromosome that affects 1 in 2500 girls. TS increases the risk of autoimmune diseases, including Graves’ disease (GD). Moyamoya disease is a rare cerebral arteriopathy of unknown etiology characterized by progressive bilateral stenosis of the internal carotid artery and its branches. Both TS and GD have been associated with Moyamoya. Type 2 spinocerebellar ataxia (SCA2) is an autosomal dominant cerebellar ataxia caused by a CAG repeat expansion in *ATXN2*. We present the first case of Moyamoya syndrome in a patient with a previous diagnosis of TS and GD who tested positive for SCA2 and had imaging findings compatible with an overlap of SCA2 and Moyamoya.

**Case presentation:**

A 43-year-old woman presented with mild gait imbalance for 2 years. Her family history was positive for type 2 spinocerebellar ataxia (SCA2). She had been diagnosed with Turner Syndrome (45,X) and Graves disease three years before. Brain MRI revealed bilateral frontal and parietal cystic encephalomalacia in watershed zones, atrophy of pons, middle cerebellar peduncles and cerebellum. MR angiography showed progressive stenosis of both internal carotid arteries with lenticulostriate collaterals, suggestive of Moya-Moya disease. Molecular analysis confirmed the diagnosis of SCA2.

**Conclusions:**

With increased availability of tools for genetic diagnosis, physicians need to be aware of the possibility of a single patient presenting two or more rare diseases. This report underscores the modern dilemmas created by increasingly accurate imaging techniques and available and extensive genetic testing.

## Background

Turner syndrome (TS) is a rare condition associated with a completely or partially missing X chromosome that affects 1 in 2500 girls. It is characterized by hypergonadotropic hypogonadism, infertility, short stature, endocrine and metabolic disorders, as well as other medical conditions. Bicuspid aortic valve is the most common congenital heart malformation, but other cardiovascular diseases also occur. Adult women are at increased risk of hypertension, stroke, coronary artery disease, heart failure and aortic dissection [1].

Moyamoya disease is a rare cerebral arteriopathy of unknown etiology characterized by progressive bilateral stenosis of the intracranial internal carotid artery and its branches, with the concomitant development of an abnormal basal meshwork of collateral vessels that resemble a “puff of smoke” [2]. Moyamoya syndrome shows a similar angiographic pattern but is associated with different diseases and risk factors, such as neurofibromatosis type 1, autoimmune diseases, previous radiation therapy, Down syndrome, and TS [2, 3]. Diagnostic criteria for Moyamoya [4] are shown in Table 1.

**Table 1:** Diagnostic criteria for Moyamoya disease (2021)

**A. Radiological Findings**
Radiological examination such as cerebral angiography is essentially mandatory for diagnosis, and at least the following findings must be present
Especially in the case of unilateral lesions or lesions complicated by atherosclerosis, it is essential to perform cerebral angiography to exclude other diseases
**1. Cerebral angiography**
**(1) Stenosis or occlusion in the arteries centered on the terminal portion of the intracranial internal carotid artery**
**(2) Moyamoya vessels (abnormal vascular networks) in the vicinity of the occlusive or stenotic lesions in the arterial phase**
Note: Both bilateral and unilateral cases can be diagnosed as Moyamoya disease
**2.MRI and MRA**
Moyamoya disease can be diagnosed when all of the following findings are found on MRI and MRA (time-of-flight; TOF) using a scanner with a static magnetic field strength of 1.5 Tesla (T) or higher (3.0 T is even more useful)
**(1) Stenosis or occlusion of the terminal portion of the intracranial internal carotid artery**
**(2) Decrease in the outer diameter of the terminal portion of the internal carotid artery and the horizontal portion of the middle cerebral artery bilaterally on heavy T2-weighted MRI**
**(3) Abnormal vascular networks in the basal ganglia and/or periventricular white matter on MRA**
Note: When two or more visible flow voids are present in the basal ganglia and/or periventricular white matter at least unilaterally on MRI, they can be judged as representing abnormal vascular networks
Note: It is important to confirm the presence of a decrease in the outer diameter of the involved arteries on heavy T2-weighted MRI in order to differentiate atherosclerotic lesions
**B. Differential Diagnosis**
Moyamoya disease is a disease of unknown etiology, and similar cerebrovascular lesions associated with the following should be excluded as quasi-moyamoya disease or moyamoya syndrome
**(1) Autoimmune disease (SLE, antiphospholipid syndrome, polyarteritis nodosa, Sjögren syndrome, etc.),**
**(2) Meningitis,**
**(3) Brain tumors,**
**(4) Down’s syndrome,**
**(5) Neurofibromatosis type 1,**
**(6) Cerebrovascular lesions after head irradiation**
Note: Cases with hyperthyroidism can be diagnosed as moyamoya disease
Diagnostic Assessment
Moyamoya disease is diagnosed when (1) and (2) of A-1 or (1) to (3) of A-2 are met and B is excluded

TS increases the risk of autoimmune diseases. The most common are autoimmune thyroid diseases, including Graves' disease (GD), characterized by circulating autoantibodies to the thyroid-stimulating hormone receptor, leading to a hyperthyroid state. The presence of Moyamoya in Graves disease has been described in a case series of Latin American patients [3].

Type 2 spinocerebellar ataxia (SCA2) is one of the autosomal dominant cerebellar ataxias and is caused by a CAG repeat expansion in *ATXN2* resulting in progressive cerebellar ataxia and other neurological signs and symptoms, including ocular motor abnormalities. There are no previous studies reporting the association of SCA2 and Moyamoya.

We present a case of Moyamoya syndrome in a patient with a previous diagnosis of TS and GD who also had a diagnosis of SCA2 and showed imaging findings compatible with an overlap of SCA2 and Moyamoya.

## Case presentation

A 43-year-old woman presented with mild gait imbalance for 2 years. She was able to walk independently without support and did not report falls. On neurological examination there was bilateral proptosis without ophthalmoparesis, inability to assume tandem stance and ataxic gait. Deep tendon reflexes were hyperactive bilaterally, Hoffmann and Babinski signs were present. Slow and dysmetric horizontal and vertical saccades were observed, as well as mild gaze-evoked nystagmus.

**Fig. 1:**
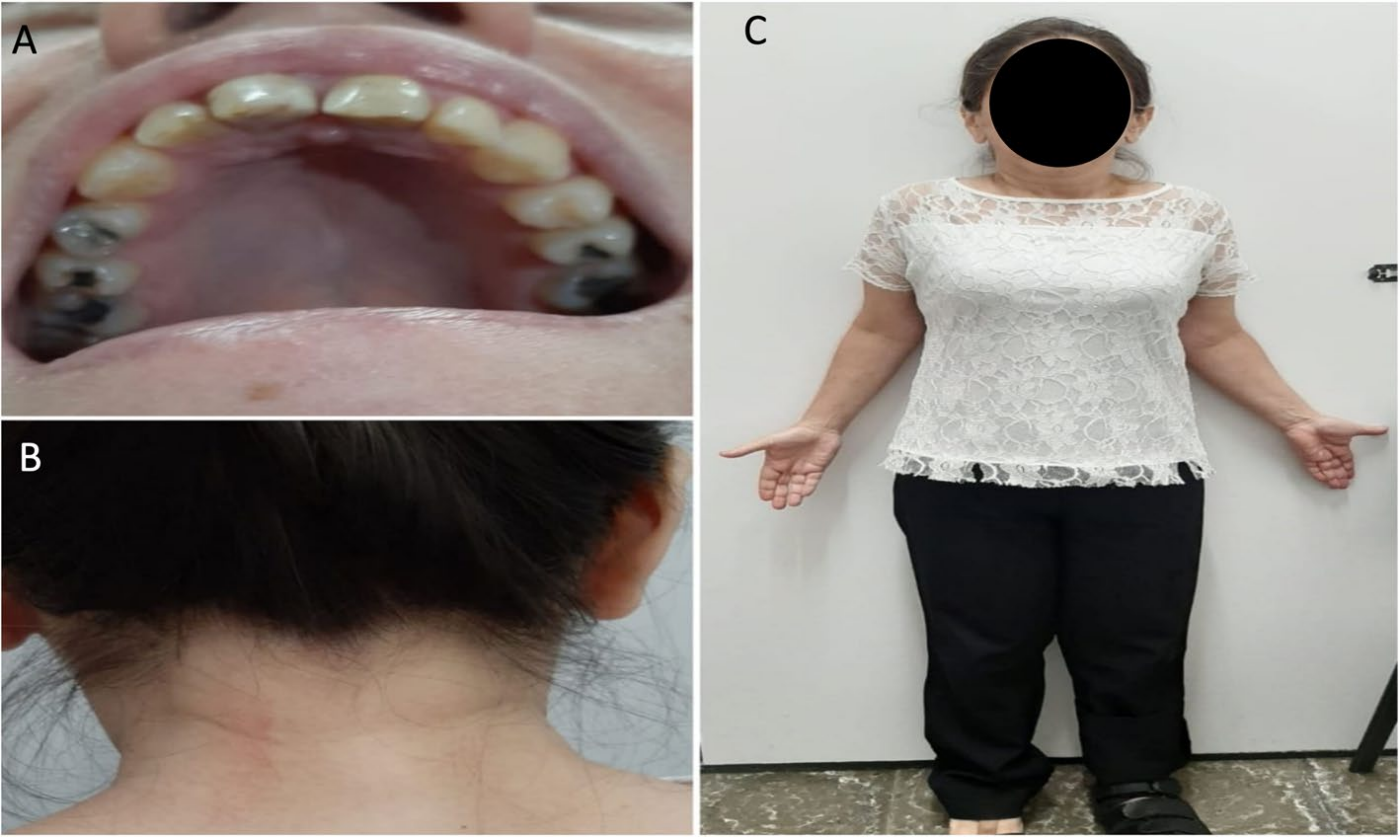
Clinical features of Turner syndrome including: **A** High-arched “ogival” palate. **B** Low “trident” hairline. **C** Short stature, low set ears, short neck and cubitus valgus

She had been diagnosed with Turner Syndrome (45,X) three years before (Fig. 1), and also had a diagnosis of Graves disease, premature ovarian failure and anxiety disorder. Her family history was positive for type 2 spinocerebellar ataxia (SCA2) (father, sister and a paternal aunt). A probable diagnosis of SCA2 was suspected based on clinical findings and family history and a brain MRI scan was ordered along with genetic testing for SCA2 by polymerase chain reaction (PCR).

**Fig. 2:**
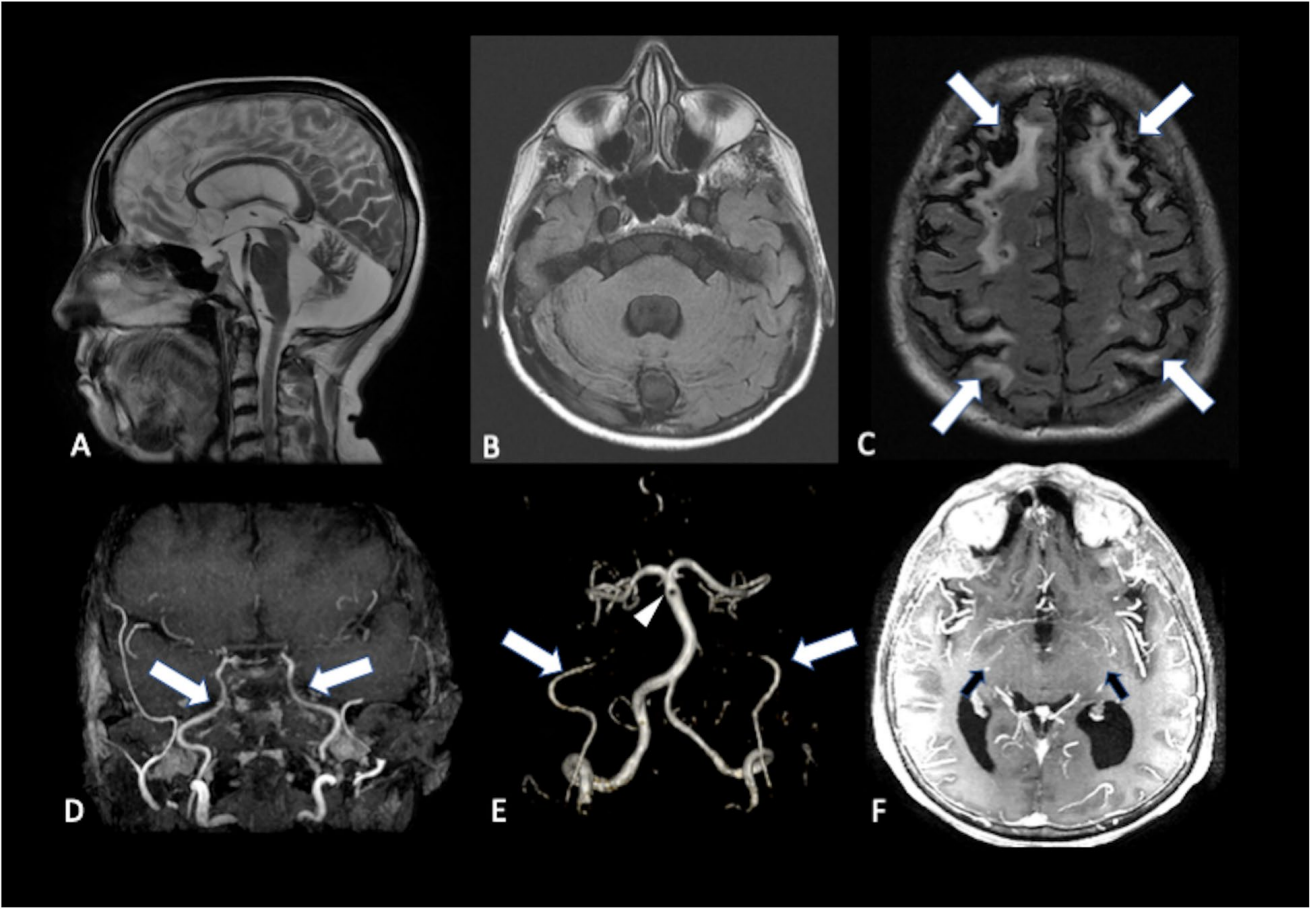
Sagittal T2 (**A**) and axial FLAIR (**B**) images showing cerebellar atrophy and (**C**) chronic watershed zone infarcts (arrows). MR angiography reveals progressive bilateral intracranial carotid narrowing, followed by occlusion in supraclinoid segments (**D**), absence of flow signal in both supraclinoid carotid segments (**E**) and abnormal net-like vessels resembling a "puff of smoke” (**F**)

Brain MRI (Fig. 2) revealed extensive bilateral frontal and parietal cystic encephalomalacia in watershed zones, atrophy of pons, middle cerebellar peduncles and cerebellum. Molecular analysis of *ATXN2* revealed a 36 CAG (normal < 32) repeat expanded allele, confirming the diagnosis of SCA2. We supposed that the cerebellar and brainstem atrophy could be explained by SCA2, but we did not believe that the bilateral chronic watershed infarcts were related to this disease, and so we ordered magnetic resonance angiography (MRA) to investigate the pathogenesis of the supposedly vascular lesions. MRA showed progressive stenosis of both internal carotid arteries with lenticulostriate collaterals, suggestive of Moyamoya disease, which provided an explanation for the bilateral watershed infarcts.

## Discussion and conclusions

To the best of our knowledge, this is the first report of an unusual association of four rare diseases: Moyamoya, SCA2, TS and GD. SCA2 is an autosomal dominant cerebellar ataxia which is very common in Cuba and also some parts of Brazil [5, 6]. Imaging findings include atrophy of cerebellum, medulla, pons, and superior cerebellar peduncles with volume loss in basal ganglia (thalamus, putamen, and pallidum) and corpus callosum [7].Supratentorial white matter lesions have not been reported in SCA2, but there are reports of supratentorial involvement in SCA3 and ataxia-telangiectasia [8, 9]. The bilateral “watershed” pattern in our patient suggested chronic carotid disease, which led us to perform a MR-angiography, which confirmed Moyamoya. These unusual imaging findings represented an overlap between Moyamoya and SCA2.

Our patient had four simultaneous rare diseases. Prevalence of SCA2 has been estimated to be 1.5/100,000, incidence of Turner syndrome of 5.5/100.000 live births and incidence of Moyamoya has been estimated to be 0.035/100.000 [10]. Graves disease has an annual incidence of 20–50/100.000, which is higher in TS patients [11]. Thus, the independent co-occurrence of these four diseases in the same patient would be highly unlikely. It is probable that at least some of them are linked by common physiopathological mechanisms.

Turner syndrome has been known to be associated with systemic vasculopathy, predominantly diffuse arterial dilation and intimal-media thickening, including some reports of Moyamoya [2, 12]. Diffuse intimal–medial thickening (IMT) in TS has been associated with estrogen deficiency in a study that compared women with TS, primary amenorrhea (PA) and controls and showed that TS and PA had similar IMT, reinforcing a role for estrogen deficiency. However, arterial dilation was not present in PA and was present in TS, being independent from estrogen deficit [13].

A review of all cases of aortic dissection in TS has found histological evidence of cystic medial necrosis in aortic tissue taken from patients with Marfan syndrome and TS, suggesting a common etiology for vascular abnormalities in these diseases [14]. This finding has led the authors to hypothesize that upregulation of matrix metalloproteinases and transforming growth factor beta (TGF-b) may lead to smooth muscle proliferation and disruption of the media, such as has been reported in Marfan [15].

Thyroxine toxicity and endothelial inflammation have been proposed as mechanisms for Moyamoya in GD [3]. Previous studies have hypothesized that disturbed cerebrovascular autoregulation in hyperthyroidism, vasospasm brought on by sympathetic nervous system activation, and changes in cerebral hemodynamics may be pathological mechanisms by which cerebral ischemia of Moyamoya syndrome deteriorates in a state of thyrotoxicosis [16, 17]. A study with 30 patients with intracranial artery stenoses has found stenotic lesions in the terminal portion of the internal carotid artery (ICA) in all patients with GD or elevated thyroid antibody levels [18] and suggested a correlation between immune-mediated thyroid diseases and stenosis of the terminal ICA (as was the case in the patient reported here). Additionally, a recent study revealed that patients with Moyamoya disease who did not have thyroid illness usually displayed high thyroid antibody levels [19]. It is still unclear how thyroid antibodies could lead to arterial stenosis.

Multiple factors may have contributed to this patient´s arteriopathy. Since many genetic and clinical conditions are associated with Moyamoya, the cause-and-effect relationship between Moyamoya and specific diseases is difficult to establish. The actual frequency of association between these diseases is difficult to estimate, but we have found 4 previous reports of Moyamoya in TS patients [2, 12, 20, 21], as well as 78 reports of Moyamoya associated with Graves disease. Moreover, Graves disease occurs in up to 1.7% of patients with Turner syndrome. Therefore, we believe that at least TS, GD and Moyamoya are not independent events and have a pathological link.

Given the evidence for a mechanistic association of TS, GD and Moyamoya, bayesian inference suggests two possible hypotheses [22]. First, SCA2 is an independent event, and the probability of such an occurrence would be estimated by multiplying the prevalence of SCA2 (1.5/100,000) by that of TS + GD + moyamoya (which is hard to estimate, as there are no previous reports of this particular association). In the second hypothesis SCA2 would share mechanisms with Moyamoya. In that case we would have to study possible correlations between SCA2 and systemic vasculopathy to look for a link. To the best of our knowledge, no previous reports of vasculopathy associated with SCA2 have been reported. This is probably a fortuitous association, given the rarity of reports and absence of a proposed pathophysiological mechanism for vasculopathy in SCA2. Moreover, TS and SCA2 are definitely independent events, as TS is a de novo disease and SCA2 is inherited, and TS is known to be associated with Moyamoya.

The main limitation of this study is the fact that it is a single case report and, as such, is not able to establish clear associations between these diseases.

With increased availability of tools for genetic diagnosis, including Next-Generation Sequencing (NGS) and commercial spinocerebellar ataxia panels, physicians need to be aware of the possibility of a single patient presenting two or more rare diseases, which might be associated with the same mutation or not, as was the case in our patient. This report underscores the modern dilemmas created by increasingly accurate imaging techniques and availability of extensive genetic testing. It is indeed a brave new world, and some cases may not adhere to Occam’s razor, but to Hickam’s dictum: patients may have as many diseases as they please [23].

## Data Availability

All materials and data used in this study will be made available upon request.

## Abbreviations

GD: Graves’ Disease
MD: Moyamoya Disease
SCA: Spinocerebellar Ataxia
SCA2: Type 2 Spinocerebellar Ataxia
ST: Turner syndrome
